# Dendritic cell‐specific hypoxia‐inducible factor‐1α deficiency protects mice from recurrent retroviral infection

**DOI:** 10.1113/EP093450

**Published:** 2026-07-05

**Authors:** Timm Schreiber, Yvonne Hüsecken, Claudia Lodewick, Simone Schimmer, Gennadiy Zelinskyy, Ulf Dittmer, Kathrin Sutter, Joachim Fandrey, Sandra Winning

**Affiliations:** ^1^ Institute of Physiology and Pathophysiology and Center for Biomedical Education and Research (ZBAF) University of Witten/Herdecke Witten Germany; ^2^ Institute of Physiology University of Duisburg‐Essen Essen Germany; ^3^ Institute for Virology University of Duisburg‐Essen Essen Germany

**Keywords:** dendritic cells, Friend virus infection, hypoxia, hypoxia‐inducible factor‐1α, retrovirus

## Abstract

Antigen‐processing and ‐presenting cells play a pivotal role in initiation of the antiviral immune response. The cellular adaption of dendritic cells to reduced oxygen and nutrition levels at the site of infection is regulated predominantly by the transcription factor complex hypoxia‐inducible factor‐1 (HIF‐1). For a better understanding of the influence of dendritic HIF‐1 on the outcome of a retroviral infection, we infected wild‐type and CD11c‐specific *HIF‐1α* knockout mice with the murine Friend leukaemia virus (FV) to cause an acute to chronic infection. Chronic FV infection caused a much more severe clinical course in control mice than in knockout mice, although no gross differences in spleen weight and immune cell population during acute FV infection were observed. Following chronic FV infection, half of the control mice developed massive splenomegaly accompanied by a complete loss of splenic architecture, whereas the remaining control mice appeared able to control viral replication. In contrast, without exception all knockout mice were able to restrict viral replication. Furthermore, control mice showed increased numbers of antigen‐presenting cells with impaired activation and decreased adaptive immune response. Given that our knockout construct leads to an HIF‐1α protein without a functional DNA binding domain, and it was already shown that HIF‐1α plays a non‐transcriptional role in DNA replication, we analysed basal *HIF‐1α* mRNA expression. All animals with a very low basal *HIF‐1α* expression incurred FV recurrence, whereas higher basal expression was protective against recurrence. These results might indicate a novel role of HIF‐1α in control of chronic viral infections.

## Abstract (highlights)

- **What is the central question of this study?**
  We examined the role of dendritic cell‐specific hypoxia‐inducible factor‐1α (HIF‐1α) for the recurrence of Friend virus (FV) causing severe murine splenomegaly despite initial recovery from acute FV infection.
- **What is the main finding and its importance?**
  Dendritic cell‐specific HIF‐1α determined whether FV infection resolved or progressed to recurrence with splenomegaly. Wild‐type mice sometimes developed recurrent infection with severe splenomegaly, immune suppression and viral rebound, whereas mice lacking HIF‐1α in dendritic cells never showed recurrence and recovered after the acute phase. Protection was linked to higher basal *HIF*‐1α expression, potentially interfering with viral replication.

## 1 INTRODUCTION

The murine Friend leukaemia virus (FV) is a retroviral complex, which causes lethal erythroleukaemia and splenomegaly in susceptible mice. It consists of a replication‐competent (but, in adult mice, non‐pathological) helper virus, called Friend murine leukaemia virus and the replication‐defective but pathogenic spleen focus‐forming virus (Halemano et al., 2013; Hasenkrug & Chesebro, 1997). Only co‐infection of target cells with both viruses leads to a successful replication cycle of spleen focus‐forming virus. The envelope glycoprotein gp55 of spleen focus‐forming virus binds to the erythropoietin receptor of erythrocyte progenitor cells and induces a massive erythropoietin‐independent expansion of these cells, which is the major reason for the enlarged spleen during the course of infection (Kabat, 1989; Moreau‐Gachelin, 2008). FV is also able to affect the immune system by infection of granulocytes, monocytes and lymphocytes (Dittmer et al., 2002). However, mice belonging to disease‐resistant strains, such as C57Bl/6, are normally able to recover from FV infection after developing a strong immune response during the acute phase of infection (Hoatlin & Kabat, 1995; Lilly, 1968). As a first line of defence, the specialized antigen‐processing and ‐presenting dendritic cells (DCs) play a pivotal role in the initiation of the antiviral immune response (Banchereau & Steinman, 1998). Antigen or cytokine contact of DCs leads to maturation and upregulation of co‐stimulatory molecules and major histocompatibility complexes (MHCs). Following immigration into T‐cell regions of lymph nodes and the spleen, activated DCs present the peptide–MHC complex to CD4^+^ and CD8^+^ T cells, leading to lymphocyte activation (Cravens & Lipsky, 2002; Schmid et al., 2010). Reduction of viral load and recovery from FV infection is driven mainly by an adequate antigen‐specific response, inducing cytotoxic activity of CD8^+^ T cells (Robertson et al., 1992). Activation and maturation of DCs, either in viral infections or in inflammatory conditions, normally takes places in conditions of low oxygen (hypoxia) and nutrients. Oxygen‐ and nutrient‐consuming immune cells invade the respective tissue, and lymph nodes show characteristically low oxygen levels. DCs have to adapt to these conditions to maintain their functional abilities (Cramer & Johnson, 2003; Cramer et al., 2003).

Cellular adaptation to hypoxia or inflammation is regulated by the transcription factor complex hypoxia‐inducible factor‐1 (HIF‐1), which is composed of an O_2_‐sensitive α‐subunit and an O_2_‐independent nuclear β‐subunit (Wang et al., 1995). In normoxic conditions (normoxia), prolyl hydroxylases continuously degrade the α‐subunit, whereas hypoxia leads to stabilization of HIF‐1α. After translocation into the nucleus, HIF‐1α dimerizes with the β‐subunit and forms the transcriptionally active HIF‐1 (Wenger, 2002). In addition, both inflammatory cytokines and viral or bacterial stimuli are able to increase *HIF‐1α* levels via Toll‐like receptor signalling even in normoxia. In DCs, bacterial stimulation can enhance *HIF‐1α* mRNA and HIF‐1α protein expression, which leads to induced expression of type I interferons (Wobben et al., 2013). Viral infections can have different influences on stabilization and activation of HIF‐1α in their target cells. Enhanced HIF‐1α during a respiratory syncytial virus infection leads to increased permeability of the lung epithelium and facilitates viral spread (Kilani et al., 2004), whereas HIF‐1‐dependent interferon production limits vesicular stomatitis virus infection (Naldini et al., 1993). In contrast, inhibition of HIF‐1 activity during the course of a vesicular stomatitis virus infection leads to enhanced cytotoxicity and viral replication (Veer et al., 2001).

In this study, we investigated the influence of HIF‐1α on DCs during acute or chronic retroviral infection by using mice with a DC‐specific *HIF‐1α* knockout.

## 2 MATERIALS AND METHODS

### 2.1 Ethical approval

The investigators understand the ethical principles under which *Experimental Physiology* operates. Our work complies fully with the animal ethics policy of the journal and with the ARRIVE 2.0 guidelines.

All mice used in this study were kept and treated in strict accordance with the German law for animal welfare and institutional regulations for animal handling of the Society for Laboratory Animal Science (GV‐SOLAS) and the European Health Law of the Federation of Laboratory Animal Science Associations (FELASA). Protocols were approved by the North Rhine‐Westphalia State Agency for Nature, Environment and Consumer Protection (LANUV; permit number: 84‐02.04.2011.A247). Animals had free access to food and water throughout the whole experimental procedure. They were housed in a constant 12 h day–12 h night cycle in specific pathogen‐free conditions. Mice were checked daily by the investigators and only suffered mildly from the experimental procedures. All experimental animals were killed humanely (cervical dislocation) after 12 weeks. Animals that were not subjected to an experiment before were killed humanely by cervical dislocation for a scientific purpose according to §4 point 3 of the German law for animal welfare.

### 2.2 Mice

**FIGURE A1:**
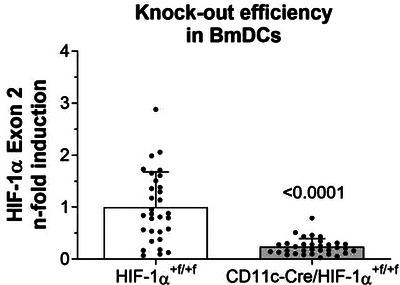
Exon 2 of *HIF‐1α* is significantly reduced in bone marrow‐derived dendritic cells (BMDCs) from knockout mice. BMDCs were isolated from naive mice and cultured in standard cell culture conditions. mRNA was isolated, and real‐time PCR was used to determine the expression of *HIF‐1α* exon 2 mRNA (mean + SD; *n* = 31–33 animals; two‐tailed, non‐parametric *t*‐test, *P* < 0.0001).

All mice were originally distributed from The Jackson Laboratory (via Charles River Germany). Double‐floxed *HIF‐1α* mice (*HIF‐1α* ^+f/+f^; RRID: IMSR_JAX:007561) on a C57Bl/6 background (Hasenkrug et al., 1998), carrying loxP sites around exon 2 of the *Hif1a* gene on both alleles, and double‐floxed *HIF‐1α* mice transgenic for CD11cCre (CD11cCre^+/ki^/*HIF‐1α* ^+f/+f^, herein shortened to *HIF‐1α* ^−/−^; RRID: IMSR_JAX:008068) (Hasenkrug & Chesebro, 1997) were included in this study. To ensure the greatest genetic overlap between the mice, breeding occurred with heterozygous Cre‐expression: Cre‐positive (*HIF‐1α* ^−/−^) and Cre‐negative (*HIF‐1α* ^+f/+f^) littermates were used. Knockout efficiency in isolated bone marrow‐derived DCs (BMDCs) of *HIF‐1α* ^−/−^ exceeded 75% (Figure A1). DCs isolated from the spleen showed a comparable knockout efficiency (data not shown). Mice (independent from Cre expression) were fertile and showed normal behaviour. Mice were randomly distributed into the control or the Friend virus‐treated group. All mice in both treatment groups were 8–16 weeks of age at the beginning of the experiments and were kept in specific pathogen‐free conditions. To avoid stressing the animals owing to the housing conditions, the animals were never kept individually in cages. This has the incidental effect that a high degree of assimilation of the microbiome of the experimental animals within a cage can be assumed. Experiments were done using mice with a C57BL/6 background resistant to FV‐induced disease (Chomczynski & Sacchi, 1987; Hasenkrug & Dittmer, 2007). None of the manipulations required anaesthesia of the animals.

### 2.3 Description of *HIF‐1α* knockout

**FIGURE A2:**
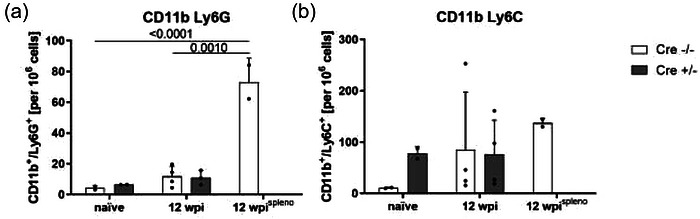
Characterization of the origin of myeloid‐derived suppressor cells. Spleens of *HIF‐1α* ^+f/+f^ and *HIF‐1α* ^−/−^ mice were isolated, and subpopulations of spleen cells were analysed by flow cytometry. The following antibodies were used for cell population analysis: (a) CD11b^+^/ MHCII^−^/Ly6G^+^, for myeloid suppressor cells of granulocytic origin; and (b) CD11b^+^/MHCII^−^/Ly6C^+^, for myeloid suppressor cells of monocytic origin. CD11b^+^/MHCII^−^/Ly6G^+^ cells were significantly induced in spleens of *HIF‐1α* ^+f/+f^ and 12 wpi^spleno^ mice compared with naive *HIF‐1α* ^+f/+f^ (*P* < 0.0001) and 12 wpi mice (*P* = 0.001). Data were analysed with ANOVA and Tukey's multiple comparison test (mean + SD). *n* = 2–4 animals.

Crossbreeding of *HIF‐1α* ^+f/+f^ mice (RRID: IMSR_JAX:007561) with mice transgenic for CD11cCre (RRID: IMSR_JAX:008068) revealed very stable Cre activity in DCs, leading to a constant excision of the exon 2 of the *HIF‐1α* mRNA (Figure A2). Excision of exon 2 resulted in expression of a shortened HIF‐1α protein, lacking the DNA binding domain, in *HIF‐1α* ^−/−^ mice. Herein, the HIF transcription factor is therefore non‐functional. Nonetheless, non‐transcriptional functions of the shortened protein are assumed to be intact.

### 2.4 Virus and viral infection

Virus stock solution used in these experiments contained an FV complex of B‐tropic Friend murine leukaemia virus and polycythaemia‐inducing spleen focus‐forming virus (Lilly & Steeves, 1973). Stock solution was prepared as a 15% spleen cell homogenate from BALB/c mice infected 14 days previously with 3000 SFFU of uncloned virus (Hasenkrug et al., 1998). After spleen removal, FV‐induced malignant cell clusters were stained with Bouin's solution and counted, giving the virus concentration in SFFU. Experimental *HIF‐1α* ^+f/+f^ and *CD11cCre/HIF‐1α* ^+f/+f^ mice were injected intravenously (injection into the tail vein) with sterile PBS containing 20 000 spleen focus‐forming units (SFFU) of FV for 5 or 11 days (acute infection) or 40 000 SFFU of FV for 12 weeks (chronic FV infection). Infection with FV was always performed by the same, highly experienced scientist and therefore showed a high degree of consistency.

### 2.5 Cell‐surface and intracellular staining by flow cytometry analysis

Two‐thirds of each spleen from naive and FV‐infected mice were homogenized in PBS using a 70 µm cell strainer (Falcon). For cell‐surface staining, 2 × 10^6^ spleen cells were incubated at room temperature with fluorescein‐conjugated antibodies obtained from Biolegend, unless otherwise indicated. The following antibodies were used in combination for surface phenotype analysis: anti‐CD4 (GK1.5), anti‐CD8a (53‐6.7), anti‐CD11b (M1/70), anti‐CD11c (N418), anti‐CD16/32 (93), anti‐CD19 (6D5), anti‐CD25 (PC61), anti‐CD43 (1B11), anti‐CD69 (H1.2F3), anti‐CD86 (GL‐1), anti‐F4/80 (BM8), anti‐Gr1 (anti‐Ly‐6G/Ly‐6C) (RB6‐8C5) and anti‐Ter119 (Ter‐119; Thermo Scientific). Dead cells (propidium iodide‐positive; Molecular Probes) were excluded from analysis. Before intracellular staining, cell‐surface staining was performed using fixable viability dye (Thermo Scientific) for detection of dead cells. Intracellular granzyme B (NGZB, Thermo Scientific) and Foxp3 (FJK‐16s, Thermo Scientific) were detected by intracellular staining using a Foxp3 staining kit (Biolegend). Data were acquired on a Canto II flow cytometer (BD Biosciences) from 100 000 living‐gated events per sample. All samples were compensated for the exclusion of fluorescence spillover. Analyses were done using FACSDiva software (BD Biosciences), and data are given as absolute counts per 10^6^ cells. The gating strategy used for the flow cytometric analysis is provided in Table 1.

**TABLE 1:** General gating strategy.

Cell type	Gating strategy (all propidium iodide‐negative/fixable viability dye‐negative)
Erythroblasts	Ter119^+^
Macrophages	F4/80^+^
DCs	CD11c^+^F4/80^−^
Granulocytes	Gr1^+^F4/80^−^
T_Helper_	CD4^+^CD8a^−^
CTL	CD4^−^CD8a^+^
Activated CTL (early) (late)	CD4^−^CD8a^+^CD43^+^ CD4^−^CD8a^+^CD69^+^
Activated, non‐degranulated CTL	CD4^−^CD8a^+^CD43^+^GzmB^+^
T_reg_	CD4^+^CD25^+^FoxP3^+^
MDSC (general)	CD11b^+^Gr1^+^MHCII^−^
MDSC (granulocytic origin)	CD11b^+^MHCII^−^Ly6G^+^Ly6C^−^
MDSC (monocytic origin)	CD11b^+^Gr1^+^Ly6G^−^Ly6C^+^

### 2.6 Cell culture conditions

All cell culture experiments were performed in cell culture incubators under water vapour saturation, 5% CO_2_ and 37°C. Hypoxic incubations were performed in a Ruskinn Invivo 300 workstation, allowing precise control of the relative oxygen abundance in the culture atmosphere.

### 2.7 Infectious centre assays

On the day before the end of the experiment, 2 × 10^4^ FV‐susceptible *Mus dunni* cells were seeded into each well of a six‐well tissue culture plate in RPMI 1640 medium (Gibco) supplemented with 10% fetal calf serum (Biochrom) and penicillin/streptomycin (100 units/mL, Thermo Scientific). Single‐cell suspensions of spleens from acute or chronically infected mice were plated in concentrations between 10^2^ and 10^7^ per well onto an *M. dunni* cell layer and were co‐cultivated for 3 days. After ethanol fixation, cells were stained with Friend murine leukaemia virus envelope‐specific monoclonal antibody 720 (Robertson et al., 1991) and developed with peroxidase‐conjugated goat anti‐mouse IgG and substrate to detect foci of infected cells (Dittmer et al., 1998).

### 2.8 Histology

One‐third of each spleen from naive and FV‐infected mice was fixed for 24 h in 4% paraformaldehyde, followed by paraffin embedding and cutting into 4‐µm‐sections before staining with Haematoxylin and Eosin. Whole‐spleen cross‐sections were analysed with a reflecting microscope. The percentage of germinal centres [(area of all germinal centres in the cross‐section/area of cross‐section) × 100] in the spleen cross‐section was calculated and statistically analysed.

### 2.9 Murine BMDCs

For the generation of BMDCs, the bone marrow of naive and FV‐infected mice was isolated by rinsing the bones (femur and tibia of both hindlimbs) with PBS. After centrifugation (2 min at 2.000 g), the cells were resuspended in RPMI 1640 medium supplemented with 10% fetal calf serum, l‐glutamine (2 mM, Thermo Scientific), sodium pyruvate (1 mM, Thermo Scientific), penicillin/streptomycin (100 µg/mL) and 2‐mercaptoethanol (0.05 mM, Merck). For the differentiation, 1 × 10^6^ bone marrow cells per well were seeded in a six‐well plate and treated with 1 ng/mL interleukin 4 (IL‐4) and 5 ng/mL granulocyte macrophage colony‐stimulating factor (GM‐CSF). The IL‐4 and GM‐CSF were obtained from cell supernatants of NIH 3T3‐GM‐CSF and NIH 3T3‐IL‐4 fibroblasts. Half of the medium was replaced every other day by medium with fresh IL‐4 and GM‐CSF. On day 7, the BMDCs were harvested for further experiments.

### 2.10 RNA extraction and quantitative real‐time PCR

Total cellular RNA of 2 × 10^6^ spleen cells was isolated by the acid phenol–chloroform extraction method (Chomczynski & Sacchi, 1987). Complementary DNA was synthesized from 1 µg RNA with oligo(dT) and Moloney murine leukaemia virus reverse transcriptase (Promega). Real‐time PCRs were performed in an iCycler iQ5 (Bio‐Rad), with SYBR^®^ green as the fluorescent dye (Eurogentec). The analysis of relative changes in gene expression was calculated using the ΔΔ*Ct* method (Livak & Schmittgen, 2001). The *n*‐fold changes of target gene expression were normalized to ribosomal protein as endogenous reference gene.

### 2.11 Whole‐cell lysate preparation and immunoblot analysis

For whole‐cell lysate preparation, BMDCs were lysed with 65 µL lysis buffer ([150 mM NaCl, 10 mM Tris (pH 7.9), 1 mM EDTA, 0.1% Igepal, 1× protease inhibitor cocktail (Roche Diagnostics)] for 20 min on ice. The lysates were centrifuged at 1200*g* for 5 min, and supernatants containing cellular proteins were collected and stored at −80°C. Protein concentrations of the supernatants were quantifieed using the Bio‐Rad protein assay reagent (Bio‐Rad, Munich, Germany).

Fifty micrograms of total‐cell lysates per lane was subjected to 7.5% SDS‐PAGE and transferred onto a nitrocellulose membrane (0.2 mM pore size; Schleicher & Schuell Microscience, Dassel, Germany). Anti‐mouse HIF‐1α was detected using a rabbit mAb against HIF‐1α (Cayman Chemicals, # 10006421, Biomol GmbH, Hamburg). Detection of α‐tubulin (Santa Cruz Biotechnology) served as loading control.

### 2.12 Statistical analysis

Experimental data are based on at least three independent experiments for each time point of infection. Statistical data were derived using either Student's *t*‐test or one‐way ANOVA with Bonferroni correction as a *post hoc* test (GraphPad Prism5 software; GraphPad). A *P*‐value of <0.05 was considered significant (*P < 0.05, **P < 0.01 and ***P < 0.001).

## 3 RESULTS

### 3.1 Severe splenomegaly after chronic FV infection arose only in mice with functional HIF‐1α

To determine the influence of HIF‐1α deficiency in DCs in the course of FV infection, we compared the spleen weights of wild‐type mice (*Hif‐1α* ^+f/+f^) and mice with a DC‐specific knockout of *HIF‐1α* (*Hif‐1α* ^−/−^) after acute (5 and 11 days) and chronic (12 weeks) FV infection. We created the *Hif‐1α* ^−/−^ mice by crossing mice with a double‐floxed *HIF‐1α* (exon 2 flanked by loxP sites) with mice heterozygous for Cre recombinase under control of the Cd11c promotor. Thus, *Hif‐1α* ^−/−^ animals have a dysfunctional HIF‐1α protein lacking the DNA‐binding domain (encoded by exon 2).

**FIGURE 1:**
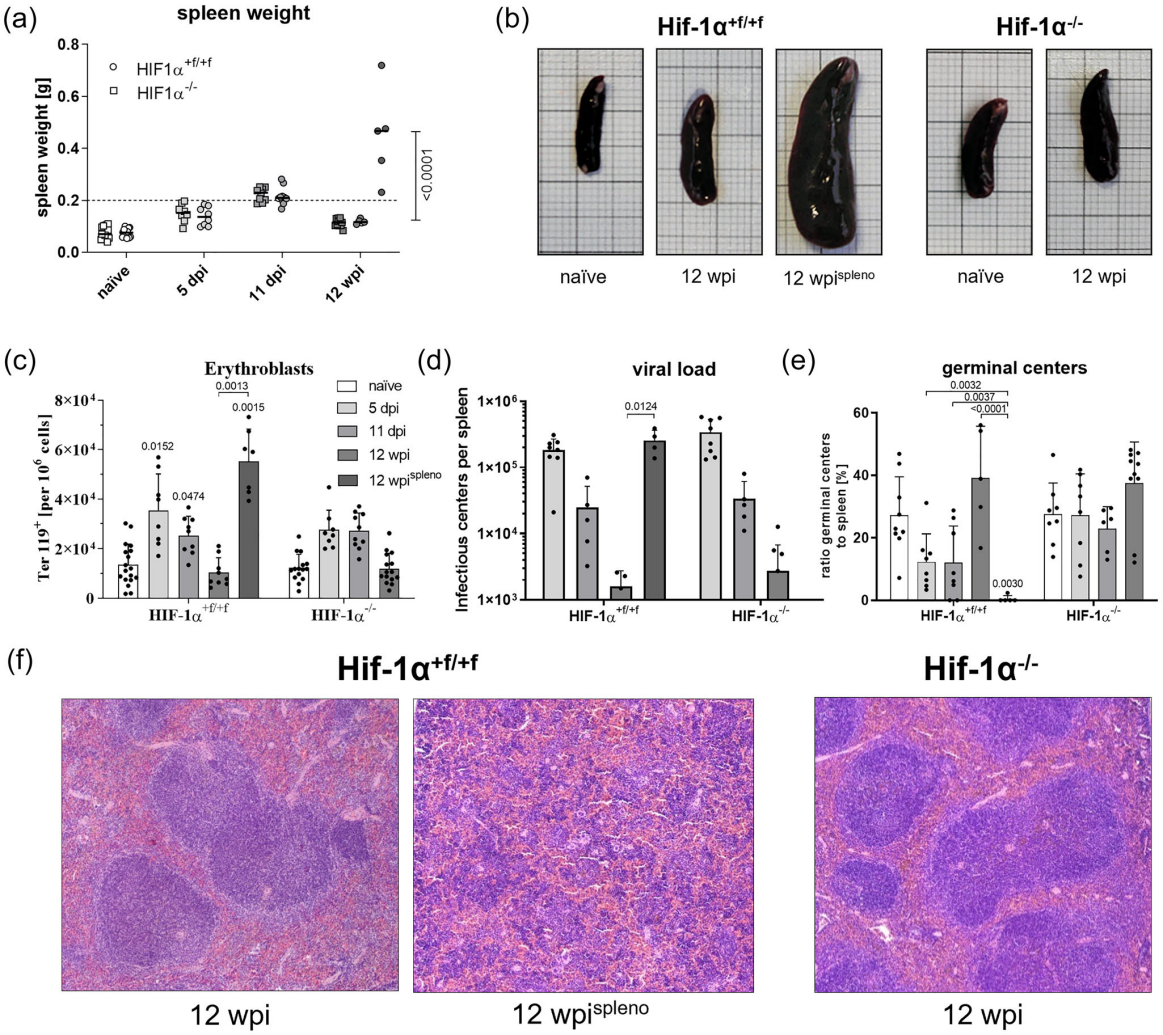
Enhanced splenomegaly after chronic FV infection of *HIF‐1α* ^+f/+f^ mice. (a) Spleen weights of hypoxia‐inducible factor (*HIF*)‐1α^+f/+f^ and *HIF‐1α* ^−/−^ mice were measured at indicated time points after FV infection. Spleen weights of half of the *HIF‐1α* ^−/−^ mice were significantly higher after 12 weeks post‐infection (*P *< 0.0001; referred to as ’12 wpi^spleno^’ hereafter). (b) Representative photomicrographs are shown. (c) The number of erythroblasts (Ter119^+^) was determined by flow cytometry and was elevated in *HIF‐1α* ^+f/+f^ mice [*P* = 0.0152 for 5 days post‐infection (dpi), *P* = 0.0474 for 11 dpi and *P* = 0.0015 for 12 wpi^spleno^ compared with naive *HIF‐1α* ^+f/+f^ and *P* = 0.0013 for the comparison of 12 wpi with 12 wpi^spleno^]. (d) Viral load was significantly elevated in *HIF‐1α* ^+f/+f^ 12 wpi^spleno^ compared with *HIF‐1α* ^+f/+f^ 12 wpi (*P* = 0.0124). (e, f) Sections of spleen tissue from naive and FV‐infected mice were stained with Haematoxylin and Eosin, and the ratio of germinal centres (GC) per spleen were determined. Acute and chronic infection of *HIF‐1α* ^+f/+f^ mice reduced the numbers of GC (*P* = 0.0032 for 5 dpi, *P* = 0.0037 for 11 dpi, *P* = 0.0030 for 12 wpi^spleno^ compared with naive *HIF‐1α* ^+f/+f^ and *P* ≤ 0.0001 for the comparison of 12 wpi with 12 wpi^spleno^). Data were analysed with ANOVA and Tukey's multiple comparison test (mean + SD). HIF: hypoxia‐inducible factor, FV: Friend Virus, dpi: days post infection, wpi: weeks post infection, spleno: splenomegaly, GC: germinal centers, SD: standard deviation.

During the course of early to late acute infection, we observed an increase of spleen weight, which was comparable in control and knockout mice (Figure 1a,b; *n* = 4–18 animals). At 12 weeks post‐infection (wpi), spleens of around half of the *Hif‐1α* ^+f/+f^ mice had developed a severe splenomegaly, whereas the other half had spleens normal in weight and size (Figure 1a,b; *n* = 4–18 animals). Interestingly, we never observed splenomegaly in any of the *HIF‐1α* ^−/−^ mice.

Owing to this distribution of the *HIF‐1α* ^+f/+f^ mice after chronic infection, we decided to analyse animals with splenomegaly separately from those that recovered from FV infection. Wild‐type and knockout mice with normal spleen size were labelled as ‘12 wpi’ and wild‐type mice with splenomegaly as ‘12 wpi^spleno^’. According to spleen size, the number of Ter119^+^ erythroblasts and erythrocyte progenitor cells increased during acute FV infection and normalized in all animals with physiological spleens after 12 wpi (Figure 1c; *n* = 4–18 animals). Significantly increased numbers of Ter119^+^ cells always accompanied the splenomegaly after 12 wpi. Our results indicate that HIF‐1α deficiency in DCs prevents mice from developing a splenomegaly in long‐term chronic infection.

Analysing viral titres during the course of FV infection, we detected that both control and knockout animals were able to limit viral replication in the course of early to late acute FV infection, but 12 wpi^spleno^ mice developed a recurrent FV infection with re‐increasing splenic viral load (Figure 1d; *n* = 4–18 animals).

It has been reported that FV infection of Balb/c, but not C57Bl/6 mice, which were used in this study, leads to a complete loss of splenic architecture within 14 days (Hegde et al., 2009). To determine the influence of dendritic loss of functional HIF‐1α on spleen structure in FV‐infected animals, we analysed Haematoxylin‐ and Eosin‐stained spleen sections of naive and FV‐infected mice. All infected *HIF‐1α* knockout mice showed only minor changes in the percentage of germinal centres and no structural alterations in splenic architecture during acute or chronic FV infection (Figure 1e; *n* = 4–18 animals). In contrast, in wild‐type mice the percentage of germinal centres decreased distinctly after acute infection. Furthermore, the splenomegaly, which developed in half our wild‐type animals, was accompanied by a complete loss of germinal centres and spleen architecture (Figure 1e; *n* = 4–18 animals). Taken together, we showed that reactions to acute infection were similar in *HIF‐1α* ^+f/+f^ and *HIF‐1α* ^−/−^ mice. However, after 12 weeks half the wild‐type mice showed a severe recurrence of FV, whereas no knockout animals were affected.

### 3.2 Mice with splenomegaly showed impaired activation of antigen‐presenting cells after 12 wpi

**FIGURE 2:**
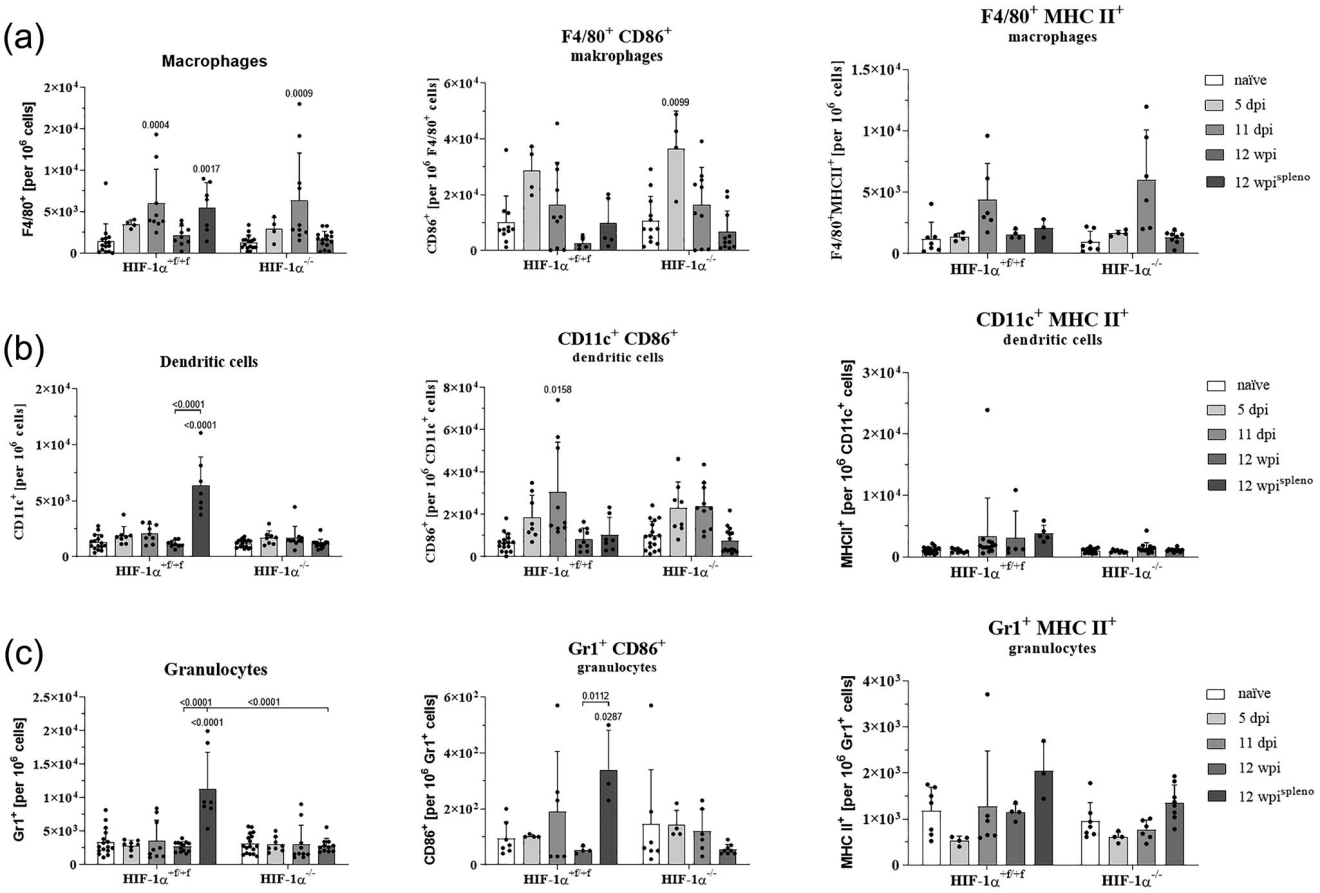
Increased antigen‐presenting cell population in *HIF‐1α* ^+f/+f^ mice at 12 wpi. Spleens from *HIF‐1α* ^+f/+f^ and *HIF‐1α* ^−/−^ mice were isolated at the indicated time points, and immune cell populations were analysed by flow cytometry. Macrophages were identified using F4/80 (a), dendritic cells using CD11c (b), and granulocytes using Gr1 (c). Activation status was assessed by surface expression of CD86 and MHCII. The number of F4/80^+^ macrophages was significantly increased in infected *HIF‐1α* ^+f/+f^ mice compared with naive controls at 11 dpi (*P* = 0.004) and in infected *HIF‐1α* ^−/−^ mice compared with naive controls at 11 dpi (*P* = 0.0009). CD11c^+^ dendritic cells were significantly increased in infected *HIF‐1α* ^+f/+f^ mice compared with naive controls at 12 wpi^spleno^ (P < 0.0001) and for 12 wpi^spleno^ compared with 12 wpi (*P* < 0.0001). Gr1+ granulocytes were significantly increased in infected *HIF‐1α* ^+f/+f^ mice compared with naive controls at 12 wpi^spleno^ (*P* ≤ 0.0001) and for 12 wpi^spleno^ with 12 wpi (*P* < 0.0001), and compared with infected *HIF‐1α* ^−/−^ mice at 12 wpi (*P* < 0.0001). CD86 expression was significantly increased in F4/80^+^ macrophages from infected *HIF‐1α* ^−/−^ mice at 5 dpi compared with naive controls (*P* = 0.0099), in CD11c^+^ dendritic cells from infected *HIF‐1α* ^+f/+f^ mice at 11 dpi compared with naive controls (*P* = 0.0158), and in Gr1*^+^* granulocytes from infected *HIF‐1α* ^+f/+f^ mice compared with naive controls at 12 wpi^spleno^ (*P* = 0.0112) and 12 wpi^spleno^ compared with 12 wpi (*P* = 0.0287). Data were analysed with ANOVA and Tukey's multiple comparison test (mean + SD). HIF: hypoxia‐inducible factor, CD: cluster of differentiation, Gr1: granulocyte‐differentiation antigen‐1, MHC: major histocompatibility complex, dpi: days post infection, wpi: weeks post infection, spleno: splenomegaly, GC: germinal centers, SD: standard deviation.

For detection of potential differences in the number of antigen‐presenting cells in FV‐infected *HIF‐1α* ^+f/+f^ and *HIF‐1α* ^−/−^ mice, we analysed the numbers of primarily F4/80^+^ macrophages, CD11c^+^ DCs and Gr1^+^ granulocytes in the spleens of naive and infected animals. We detected a continuous increase in the numbers of DCs and macrophages during the acute phase of infection, with only minor differences between wild‐type and knockout animals (Figure 2a,b; *n* = 7–15 animals). The number of granulocytes remained at control levels in both genotypes throughout the course of infection (Figure 2c; *n* = 7–15 animals). After chronic infection, the numbers of DCs and macrophages normalized in healthy animals. However, splenic macrophage numbers of 12 wpi^spleno^ mice were much higher than in naive mice and were comparable to the numbers detected at 11 days post‐infection (dpi) (Figure 2a; *n* = 7–15 animals). The splenic DC and granulocyte populations in 12 wpi^spleno^ mice showed a 4‐fold increase compared with numbers of cells in naive mice (Figure 2b,c; *n* = 7–15 animals). Although the numbers of DCs and macrophages were significantly increased in enlarged spleens at 12 wpi, only acute infection led to enhanced expression of the activation marker CD86 (Figure 2a,b; *n* = 7–15 animals). We detected the highest number of activated macrophages at 5 dpi, whereas DCs showed an increased activation throughout the course of acute infection. The number of MHCII^+^ macrophages, DCs and granulocytes did not change significantly over the entire time of infection (Figure 2a–c; *n* = 7–15 animals). Thus, enlarged spleens of 12 wpi^spleno^ mice showed an invasion especially of DCs and to a lesser extent of macrophages, with inadequate activation status as both cell types did not express CD86 on their cellular surface.

### 3.3 wpi^spleno^ mice have a reduced adaptive immune response

**FIGURE 3:**
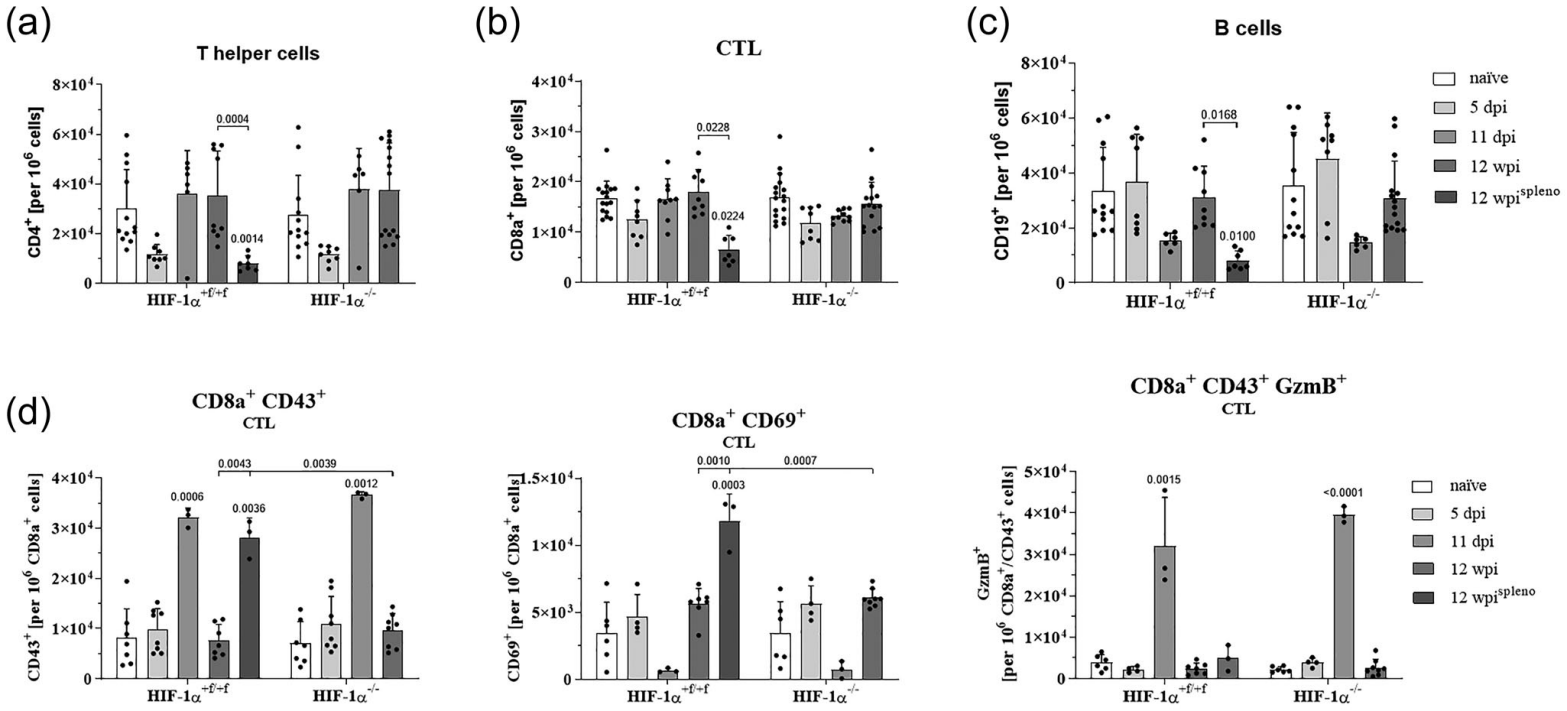
*HIF‐1α* ^+f/+f^ mice show decreased adaptive immune response at 12 weeks post‐infection. Spleens of *HIF‐1α* ^+f/+f^ and *HIF‐1α* ^−/−^ mice were isolated at the indicated time points, and subpopulations of spleen cells were analysed by flow cytometry. The following antibodies were used for cell population analysis: (a) CD4, T‐helper (T_h_) cells (significantly reduced in 12 wpi^spleno^ compared with 12 wpi, *P* = 0.0004); (b) CD8a, cytotoxic T lymphocytes (CTLs; significantly reduced in treated *HIF‐1α* ^+f/+f^ mice compared with naive *HIF‐1α* ^+f/+f^ at 12 wpi^spleno^, *P* = 0.0224, and in 12 wpi^spleno^ compared with 12 wpi, *P* = 0.0228); and (c) CD19, B cells (significantly reduced in treated *HIF‐1α* ^+f/+f^ mice compared with naive *HIF‐1α* ^+f/+f^ at 12 wpi^spleno^, *P* = 0.01, and in 12 wpi^spleno^ compared with 12 wpi, *P* = 0.0168). (d) CD43, CD69 and GzmB were used as activation markers for CTLs, showing a significantly higher CD43‐positivity of splenic CTLs from treated *HIF‐1α* ^+f/+f^ mice compared with naive *HIF‐1α* ^+f/+f^ at 11 dpi (*P* = 0.0006) and 12 wpi^spleno^ (*P* = 0.0036) and for the comparison of 12 wpi^spleno^ with 12 wpi (*P* = 0.0043). CTLs from *HIF‐1α* ^−/−^ mice also showed a significant upregulation after 11 dpi (*P* = 0.0012) but no upregulation after 12 wpi (*P* = 0.0039 for the comparison of *HIF‐1α* ^+f/+f^ 12 wpi^spleno^ with *HIF‐1α* ^−/−^ 12 wpi). Similar observations were made for CD69^+^ CTLs (*P* = 0.0003 for the comparison of naive *HIF‐1α* ^+f/+f^ and 12 wpi^spleno^; *P* = 0.001 for the comparison of *HIF‐1α* ^+f/+f^ 12 wpi^spleno^ with *HIF‐1α* ^+f/+f^ wpi, and *P* = 0.0007 for the comparison of *HIF‐1α* ^+f/+f^ 12 wpi^spleno^ with *HIF‐1α* ^−/−^ 12 wpi). Activated CTLs showed significantly higher granzyme B expression after 11 dpi independent of genotype (*P* = 0.015 for treated compared with naive *HIF‐1α* ^+f/+f^ mice and *P *< 0.0001 for treated compared with naive *HIF‐1α* ^−/−^ mice). Data were analysed with ANOVA and Tukey's multiple comparison test (mean + SD). HIF: hypoxia‐inducible factor, CD: cluster of differentiation, Gr1: granulocyte‐differentiation antigen‐1, MHC: major histocompatibility complex, dpi: days post infection, wpi: weeks post infection, spleno: splenomegaly, CTLs: cytotoxic T lymphocytes, SD: standard deviation.

The specific cellular immune response against FV is mediated by CD4^+^ T‐helper cells and CD8^+^ cytotoxic T cells. Early acute FV infection led to a decrease of CD4^+^ cells in both infected *HIF‐1α* ^+f/+f^ and *HIF‐1α* ^−/−^ mice (Figure 3a; *n* =  7–15 animals). Although the number of immune cells normalized in all animals with physiological spleens during the course of FV infection, we detected a significant decrease of CD4^+^ cells in 12 wpi^spleno^ mice. Likewise, although the distribution of CD8^+^ cytotoxic T lymphocytes (CTLs) was similar during infection between wild‐type and knockout animals, CTLs were significantly lower only in 12 wpi^spleno^ mice compared with the other animals (Figure 3a; *n* =  7–15 animals). Decreased numbers of CD19^+^ B cells were detected at 11 dpi (Figure 3a; *n* =  7–15 animals). Although the population recovered in all mice with physiological spleens, 12 wpi^spleno^ mice exhibited a further decrease of splenic B cells. Taken together, 12 wpi^spleno^ mice showed a significant decrease of splenic adaptive immune cells compared with chronically FV‐infected mice with physiological spleens.

To analyse further why only control animals developed a splenomegaly in chronic FV infection, we characterized the activation status of lymphocytes in the spleen. Early activation of T cells is characterized by an increased cell surface expression of CD69, whose downregulation is crucial for the emigration to secondary lymphoid organs (Nakayama et al., 2002). Late acute FV infection (11 dpi) led to a distinct decrease of pre‐activated splenic CD69^+^CD8^+^ T cells in both wild‐type and knockout mice, with recovery to levels of naive mice in animals without pathological findings at 12 wpi. Only 12 wpi^spleno^ mice showed a significantly increased number of CD69^+^ CTLs (Figure 3b; *n* = 7–15 animals). Additional co‐stimulatory activation of T cells establishes effector activity accompanied by an upregulation of CD43 expression (Harrington et al. 2000). We detected a significant increase of splenic CD43^+^CD8^+^ CTLs at 11 dpi, which normalized in individuals without splenomegaly at 12 wpi (Figure 3b; *n* = 7–15 animals). In contrast, the numbers of CTLs expressing CD43 in 12 wpi^spleno^ mice stayed similarly high compared with late acute infection. Cytotoxic effector CD8^+^ T cells usually express cytotoxic molecules, such as granzyme B (GzmB). GzmB is a serine protease, which is essential for the cytotoxic activity and induction of apoptosis in the target cells (Lord et al. 2003). CD43^+^ activated CTLs showed a significantly increased GzmB^+^ expression in *HIF‐1α* ^+f/+f^ and *HIF‐1α* ^−/−^ mice during late acute infection. However, 12 wpi^spleno^ mice with a splenomegaly failed to increase the number of splenic CD43^+^/GzmB^+^ CTLs (Figure 3b; *n* = 7–15 animals). Taken together, the splenomegaly observed in 12 wpi^spleno^ mice was accompanied by a defective activation of CD8^+^ CTLs with impaired cytotoxic activity.

### 3.4 wpi^spleno^ mice exhibit an immunosuppressive splenic milieu that enables FV recurrence

**FIGURE 4:**
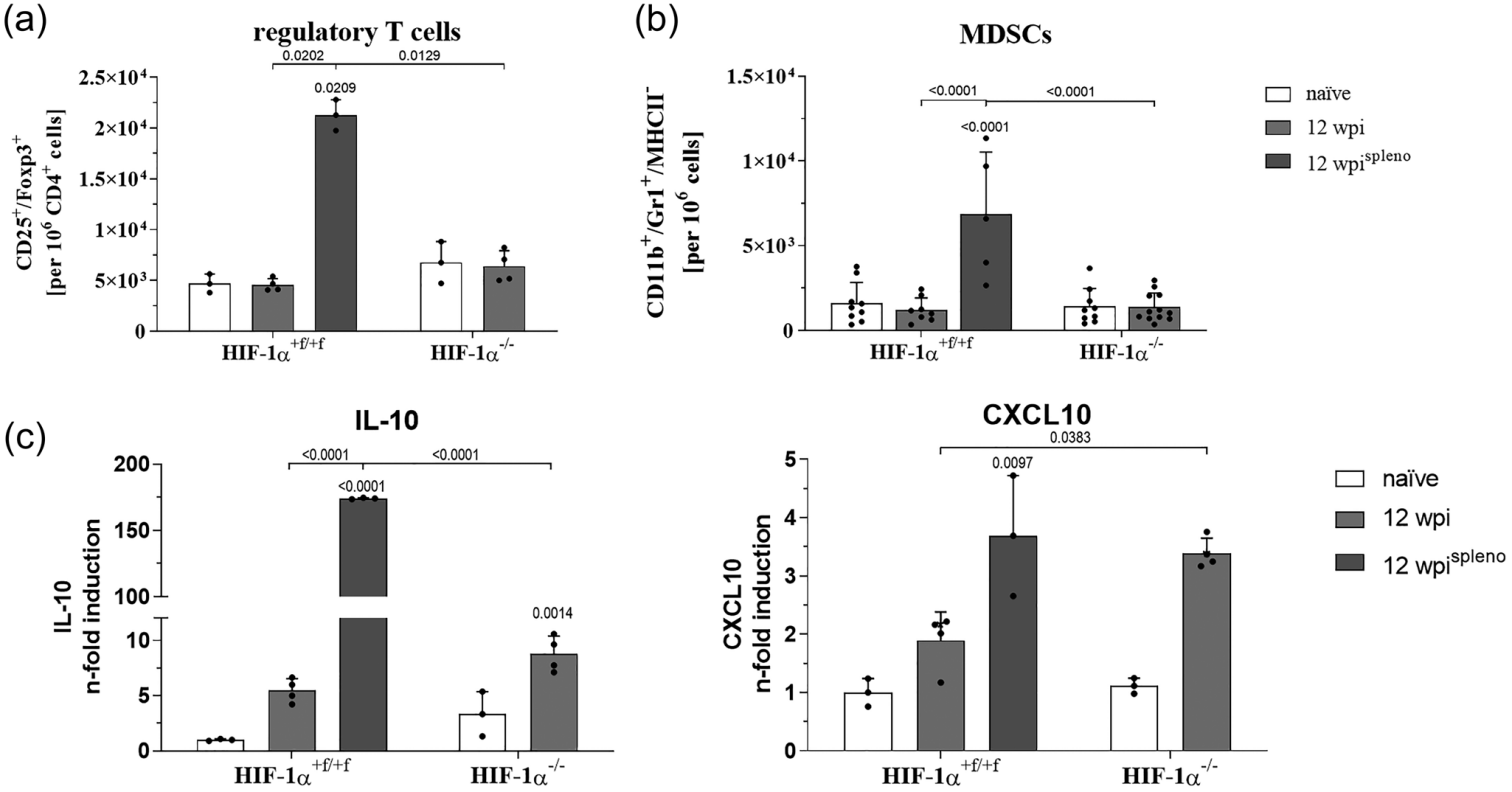
*HIF‐1α* ^+f/+f^ mice with splenomegaly at 12 wpi show immune suppression. Spleens of *HIF‐1α* ^+f/+f^ and *HIF‐1α* ^−/−^ mice were isolated at the indicated time points, and subpopulations of spleen cells were analysed by flow cytometry. The following antibodies were used for cell population analysis: (a) CD4/CD25/Foxp3, regulatory T‐helper cells; and (b) CD11b^+^/Gr1^+^/MHCII^−^, myeloid suppressor cells in spleens of naive and FV‐infected mice were removed 12 weeks after infection (wpi). Numbers of regulatory T cells were significantly induced in treated *HIF‐1α* ^+f/+f^ mice compared with naive *HIF‐1α* ^+f/+f^ and 12 wpi^spleno^ (*P* = 0.0209) and for the comparison of 12 wpi^spleno^ with 12 wpi (*P* = 0.0202) and with *HIF‐1α* ^−/−^ 12 wpi (*P* = 0.0129). Numbers of MDSCs were significantly induced in treated *HIF‐1α* ^+f/+f^ mice compared with naive *HIF‐1α* ^+f/+f^ and 12 wpi^spleno^ (*P *< 0.0001) and for the comparison of 12 wpi^spleno^ with 12 wpi (*P *< 0.0001) and with *HIF‐1α* ^−/−^ 12 wpi (*P *< 0.0001). (c) Next, mRNA was isolated, and real‐time PCR was used to determine the expression of interleukin 10 (*IL‐10*) and C‐X‐C‐motif chemokine ligand 10 (*CXCL10*). *IL‐10* mRNA expression was significantly induced in spleen cells from treated *HIF‐1α* ^+f/+f^ mice compared with naive *HIF‐1α* ^+f/+f^ and 12 wpi^spleno^ (*P *< 0.0001) and for the comparison of 12 wpi^spleno^ with 12 wpi (*P *< 0.0001) and with *HIF‐1α* ^−/−^ 12 wpi (*P *< 0.0001). *IL‐10* mRNA of spleen cells from *HIF‐1α* ^−/−^ 12 wpi mice was also significantly enhanced compared with naive *HIF‐1α* ^−/−^ (*P* = 0.0014). *CXCL10* mRNA expression was significantly induced in spleen cells from treated *HIF‐1α* ^+f/+f^ mice compared with naive *HIF‐1α* ^+f/+f^ and 12 wpi^spleno^ (*P* = 0.0097) but also elevated in *HIF‐1α* ^−/−^ 12 wpi compared with *HIF‐1α* ^+f/+f^ 12 wpi (*P* = 0.0383). Data were analysed with ANOVA and Tukey's multiple comparison test (mean + SD). HIF: hypoxia‐inducible factor, CD: cluster of differentiation, Gr1: granulocyte‐differentiation antigen‐1, MHC: major histocompatibility complex, dpi: days post infection, wpi: weeks post infection, spleno: splenomegaly, CXCL: Chemokine (C‐X‐C motif) ligand, IL: interleukin, SD: standard deviation.

At the peak of viral replication, CD4^+^ regulatory T cells (T_regs_) start to expand and suppress the antiviral activity of CD8^+^ CTLs, leading to functional impairment of CTL activity during chronic FV infection (Hasenkrug & Dittmer, 2007). To investigate the cause of our observed reduction in the adaptive immune response in the spleen, we stained spleen cells of chronically infected *HIF‐1α* ^+f/+f^ and *HIF‐1α* ^−/−^ mice for CD4^+^/CD25^+^/FoxP3^+^ T_regs_. Although the total number of CD4^+^ T cells within the population was significantly reduced in 12 wpi^spleno^ mice, the percentage of T_regs_ within the CD4^+^ T‐cell population was significantly increased in these mice (Figure 4a; *n* = 3–15 animals).

Myeloid‐derived suppressor cells (MDSCs) also have high immunosuppressive abilities and are defined as CD11b^+^/Gr1^+^/MHCII^−^ cells. They can be divided into granulocytic and monocytic MDSCs, both of which have individual immunosuppressive effects (Gabrilovich et al., 2012). From acute (data not shown) to chronic FV infections, we found only minor changes in the splenic MDSC population in both wild‐type and knockout mice (Figure 4b; *n* = 3–15 animals). However, following chronic FV infection the 12 wpi^spleno^ mice exhibited a significant increase of MDSCs, which was inversely proportional to the number of T and B cells in the spleen (Figures 3a and 4b; *n* = 3–15 animals). We found slightly higher levels of MDSCs from monocytic origin (Figure A2; *n* = 2–4 animals). The relative increase in T_regs_ and MDSCs seems to play an important immunosuppressive role in those mice that developed a splenomegaly in chronic FV infections.

Cytokines and chemokines expressed by various immune cells can further modulate inflammatory or anti‐inflammatory cytokines. Interleukin‐10 (IL‐10) is expressed by many cells of the innate and adaptive immune response, including the immunosuppressive MDSCs and T_regs_ (Saraiva & O'Garra, 2010). The 12 wpi^spleno^ mice showed a significantly increased splenic *IL‐10* mRNA expression (Figure 4c; *n* = 3–15 animals) correlating with the high amount of immunosuppressive cells (Figure 4a,b; *n* = 3–15 animals). The chemokine C‐X‐C motif chemokine ligand 10 (CXCL10) is secreted in an interferon‐γ‐dependent manner by several cell types, including T lymphocytes and myeloid cells (Antonelli et al. 2014). CXCL10 expression of DCs leads to T‐cell retention in the secondary lymph nodes, and T cells upregulate secretion of several chemokines, including CXCL10, during exhaustion in chronic viral infection (Wherry et al., 2007; Yoneyama et al., 2002). Chronic FV infection led to a significant increase of *Cxcl10* mRNA expression in the spleen (Figure 4c; *n* = 3–15 animals). The highest *Cxcl10* induction was detected in 12 wpi^spleno^ mice. Combined, 12 wpi^spleno^ mice showed an anti‐inflammatory splenic milieu that suppressed lymphocyte activity.

### 3.5 Basal *HIF‐1α* expression is involved in FV recurrence

**FIGURE 5:**
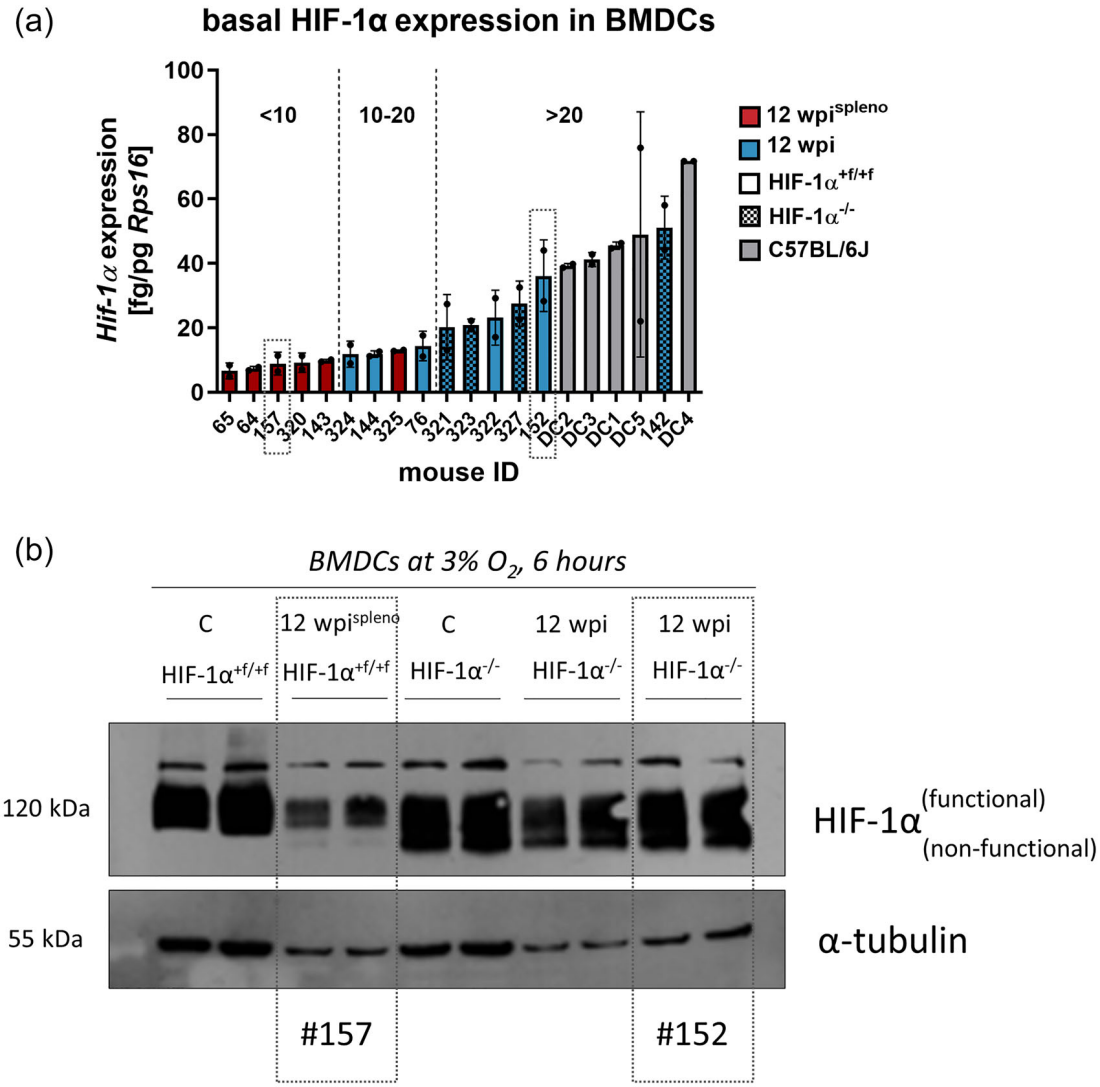
Mice with low levels of basal *HIF‐1α* expression are prone to FV recurrence. Bone marrow‐derived dendritic cells (BMDCs) were isolated from naive and FV‐infected mice at the indicated time points post‐infection and cultured in standard cell culture conditions. (a) mRNA was isolated, and real‐time PCR was used to determine the expression of *HIF‐1α*. *n* = 2 technical replicates per animal. (B) Fifty micrograms of whole‐cell protein lysates from BMDCs was subjected to immunoblot and analysed for HIF‐1α and α‐tubulin protein expression. Low basal *HIF‐1α* mRNA corresponded to low levels of protein stabilization, which were also much lower compared with that in BMDCs from naive control mice. The highlighted analysed mice in (b) are identical to the indicated ones analysed for mRNA levels in (a). HIF: hypoxia‐inducible factor, dpi: days post infection, wpi: weeks post infection, spleno: splenomegaly,Rps: ribosomal protein.

Is the suppression of lymphocyte activity the cause of FV recurrence or is it a consequence of the recurrence? Moreover, why are mice without functional HIF‐1α in DCs protected against the recurrence of FV? To address these questions, we analysed basal *HIF‐1α* mRNA expression in BMDCs of chronically infected wild‐type and knockout mice, in addition to normal C57BL/6J mice, because it was shown that HIF‐1α plays a non‐transcriptional role in DNA replication (Hubbi et al., 2013). All animals with a very low basal *HIF‐1α* expression, under 10 fg/pg *Rps16* (Ribosomal protein s16), incurred a FV recurrence, whereas in mice with an intermediate *HIF‐1α* expression (10–20 fg/pg *Rps16*), only one in four animals showed a recurrence. All animals with a basal *HIF‐1α* expression of >20 fg/pg *Rps16* were protected against FV recurrence (Figure 5a; *n* = 2 technical replicates per animal), and a low basal mRNA expression of *HIF‐1α* corresponded to less stabilized HIF‐1α protein during hypoxia in BMDCs from these mice. The general hypoxic stabilization of HIF‐1α protein (although shortened and therefore transcriptionally non‐functional in *HIF‐1α* ^−/−^ animals) did not differ between the genotypes of the mice (Figure 5b; *n* = 2 technical replicates per animal). Interestingly, all tested animals with a HIF‐1α protein lacking its DNA‐binding domain were in the group of mice with a high basal *HIF‐1α* expression in BMDCs. This might be a compensatory effect for the loss of the transcriptional function of HIF‐1 in these cells. Combined, we showed that basal *HIF‐1α* expression is correlated with the chance of FV recurrence.

## 4 DISCUSSION

In addition to cellular adaptation to hypoxia, HIF‐1 regulates the gene expression profile of DCs and macrophages in inflammatory conditions. Both bacterial and viral stimulation are able to induce a HIF‐1‐mediated immune response by activation of the Toll‐like receptor signalling pathway (Zinkernagel et al., 2007). In the present study, we used a transgenic mouse model with a CD11c‐specific *HIF‐1α* knockout to analyse the influence of dendritic HIF‐1α on the course of acute and chronic FV infection. The distribution of spleen weight, viral load and the number of erythrocytes in the spleen of infected wild‐type and knockout mice was comparable to the course of an acute infection in resistant wild‐type C57Bl/6 mice (Hoatlin & Kabat, 1995; Zelinskyy et al., 2009). Resistant mice are able to recover from FV infection within a fortnight and remain persistently infected for life with low virus levels. Earlier publications have not shown recurrent FV infections of resistant strains over a period of 90 days (13 weeks) (Chesebro et al., 1979; Hasenkrug, 1999; Zelinskyy et al., 2006). Interestingly, 12 wpi, half of the wild‐type mice developed a chronic recurrent FV infection; in contrast, this was never the case in mice with dendritic HIF‐1α deficiency, which recovered from FV infection completely. The recurrence came along with a severe splenomegaly attributable to a significant increase of erythrocytes and with re‐increasing viral load (Figure 1a–d). Additionally, we detected a total loss of splenic architecture, which was attributable to large amounts of invading erythrocytes (Figure 1e). A similar phenotype was described previously for susceptible Balb/c mice after short‐term FV infection, but not for resistant strains (Hegde et al., 2009).

The destruction of germinal centres is important for evading and inhibiting the virus‐specific immune response. During acute human immunodeficiency virus 1 (HIV‐1) infection, extensive loss of germinal centres prevents B‐cell activation and maturation initiated by CD4^+^ T cells (Levesque et al., 2009). Besides erythrocyte progenitors, FV is also able to infect different cells of hematopoietic stem cell origin, including DCs, macrophages and all other myeloid cells (Dittmer et al., 2002; Marcelletti & Furmanski, 1979; Toniolo et al., 1980; Weissman, 2002). The recurrent infection in 12 wpi^spleno^ mice led to an FV‐induced proliferation and significant increase of CD11c^+^ DCs and, to a lesser extent, of macrophages, with both cell types lacking co‐stimulatory molecule expression (Figure 2a,b). Nair et al. (2010) have shown convincingly that only activated antigen‐presenting DCs that express co‐stimulatory molecules, such as CD86, on their surface are able to prime naive endogenous CTLs to expand and to execute effector functions. Thus, it is likely that expanded DCs in 12 wpi^spleno^ mice are not able to induce sufficient T‐cell activation. During acute FV infection, cytotoxic CD8^+^ T cells are of particular relevance for the reduction of viral load because they eliminate infected cells. Depletion of CTLs in resistant mice inhibits an effective early anti‐viral immune response and leads to increased viral load (Dittmer et al., 2002). Although CD4^+^ T‐cell depletion does not inhibit the FV‐specific CTL response during the first 2 wpi, it negatively affects the CD8^+^ memory T‐cell response (Nair et al., 2010). Thus, not only does suppression of the virus‐specific CD8^+^ T‐cell response favour a chronic recurrent infection, but also decreased numbers of CD4^+^ T cells negatively affect the progress of disease. In addition to defective DC and macrophage activation, we observed a significant decrease of CD4^+^ and CD8^+^ T cells and of B cells in 12 wpi^spleno^ mice (Figure 3a). The relative decrease of lymphocytes could be attributable to the large amounts of FV target cells, such as erythrocytes, DCs and macrophages (Weissman, 2002). We detected an enhanced pre‐activation and activation of CD8^+^ CTLs in the spleen of 12 wpi^spleno^ mice. In contrast to normal T‐cell activation, enhanced CD43 expression did not involve downregulation of CD69. We also did not detect a significant increase in expression in GzmB (Figure 3b), suggesting that there was no enhanced cytotoxic activity. Contact of CTLs with the pathogen peptide–MHC I complex of DCs leads to a fast upregulation of the earliest lymphocyte marker, CD69 (Alari‐Pahissa et al., 2012; Banchereau & Steinman, 1998). But only additional binding of the ligand CD80 or CD86 to the T‐cell receptor CD28 induces maturation and rapid downregulation of CD69. Missing co‐stimulatory signalling, such as that detected on DCs after chronic recurrent FV infection in 12 wpi^spleno^ mice, leads to anergy and cell death of T cells after antigen contact (Sugamura et al., 2004; Zhang & Bevan, 2010). Promotion of CD69 upregulation on the CTL surface has been found in the presence of monocytic MDSCs and prevents T cells from migration out of lymphatic organs (Liu et al., 2011). Virus‐activated T cells, in turn, are the main producers of factors necessary for MDSC activation, such as interferon‐γ, IL‐4 and IL‐13β. Chronic persistent FV infection leads to virus‐induced increase of CD4^+^ T_reg_ cells following limitation and dysfunction of CD4^+^ and CD8^+^ T‐cell function (Zelinskyy et al., 2006, 2005). In spleens of 12 wpi^spleno^ mice, >20% of the CD4^+^ T cells expressed CD25 and FoxP3 and therefore belonged to the T_reg_ population (Figure 4a).

Interestingly, we could also detect induced levels of MDSCs in spleens of 12 wpi^spleno^ mice (Figure 4b). MDSCs have many different immunosuppressive characteristics. Nutrient depletion by activated MDSCs results in downregulation of T‐cell receptor ε‐chain following proliferation arrest of antigen‐activated T cells. Nitration and desensitizing of T‐cell receptors by reactive nitrogen species inhibit the interaction with peptide–MHC complex and the T‐cell immune response (Gabrilovich et al., 2012; Nagaraj et al., 2007). MDSCs have only a minor role during acute infection, but chronic viral infections, for example, with lymphocytic choriomeningitis virus, can inhibit differentiation of myeloid progenitor cells, inhibit maturation of myeloid cells and promote proliferation of the MDSC population (Gabrilovich & Nagaraj, 2009; Norris et al., 2013). The increase in MDSCs in 12 wpi^spleno^ mice is in inverse proportion to the lymphocyte population and might support immune suppression.

In addition, we detected a significant increase of *Il10* and *Cxcl10* mRNA expression in the spleen of control wild‐type mice with recurrent FV infection (Figure 4c,d). IL‐10 directly inhibits the proliferation and effector activity of all T cells and reduces antigen presentation and expression of pro‐inflammatory cytokines by macrophages and DCs (Liu et al., 2011; Vries, 1995; Wilson & Brooks, 2011). In chronic uncontrolled HIV infection, upregulation of IL‐10 plasma protein and mRNA expression is correlated with increasing viral load and leads to immune suppression via expansion of MDSCs (Brockman et al., 2009; Vollbrecht et al., 2012). This dependence of *Il10* mRNA expression and increase of viral load during HIV infection resembles our results in the animal model of 12 wpi^spleno^ mice. Increased *Cxcl10* expression is generally associated with chronic viral infections, which often involve severe defects in the CD8^+^ T‐cell response (Antonelli et al., 2008; Harvey et al., 2003; Liu et al., 2011). Interdependent or not, T_regs_ and MDSCs are expected to suppress lymphocyte functions. This fact is surely to be seen as a consequence of the high viral load in the spleens of the 12 wpi^spleno^ mice, but it might also contribute to the persistently high levels of FV in these mice.

But why did recurrence occur in the first place in some animals and not in others, and only in wild‐type animals, but never in mice with dendritic loss of HIF‐1α? Besides its role as a transcription factor in cellular adaptation to sudden hypoxic conditions, HIF‐1α also has non‐transcriptional roles. Hubbi et al. (2013) showed that HIF‐1α binds CDC6, a protein essential for loading the minichromosome maintenance (MCM) complex onto DNA. Binding of HIF‐1α to this complex leads to decreased phosphorylation and activation of the MCM complex. Moreover, it was shown that the MCM complex also interacts with viral RNA‐dependent RNA polymerase that is involved in viral replication. This was shown for different RNA viruses, such as influenza (Kawaguchi & Nagata, 2007) and HIV (Santos et al., 2016). Within the present study, we showed that only animals with a low basal *HIF‐1α* expression were prone to FV recurrence, whereas mice with a high basal expression, especially *HIF‐1α* knockout animals and common C57BL/6J mice, were protected against recurrence (Figure 5a; Dietze et al., 2011; Zelinskyy et al., 2009). Here, it is important to note that our *HIF‐1α* knockout construct produces an HIF‐1α protein without the DNA‐binding domain that leads to a loss of function as a transcription factor but would probably not affect any non‐transcriptional functions of HIF‐1α. Even during normal oxygen concentrations, there is a constant transcription and translation of *HIF‐1α*; if oxygen is available as a substrate, prolyl hydroxylases and the Factor Inhibiting HIF (FIH) rapidly target HIF‐1α for degradation and transcriptional inactivation. However, even in normoxic conditions, an equilibrium between translation and degradation is expected to result in a basal level of HIF‐1α protein. We observed higher amounts of HIF‐1α protein in animals with elevated basal *HIF‐1α* mRNA expression (Figure 5b). Based on this correlation, we hypothesise that increased HIF‐1α protein levels might promote binding to CDC6, potentially inhibiting the MCM complex and thereby limiting productive elongation of viral RNA. We also speculate that such a reduction in viral replication would be insufficient during acute infection with high viral loads but could be sufficient to support immune‐mediated viral control during chronic infection and thus might contribute to the prevention of viral recurrence.

## 5 CONCLUSION

In summary, we found that chronic FV infection leads to a recurrence with a massive splenomegaly only in wild‐type mice, but never in mice with a dendritic *HIF‐1α* knockout. This is attributable to a lower basal *HIF‐1α* expression in wild‐type animals that we assume to prevent productive viral cRNA elongation by inhibition of the MCM complex.

## AUTHOR CONTRIBUTIONS

Kathrin Sutter, Ulf Dittmer, Sandra Winning and Joachim Fandrey conceptualized and designed the study. Data acquisition was performed by Yvonne Hüsecken, Claudia Lodewick, Simone Schimmer and Timm Schreiber. Data analysis and interpretation were performed by Yvonne Hüsecken, Timm Schreiber, Kathrin Sutter, Gennadiy Zelinskyy and Sandra Winning. Timm Schreiber and Sandra Winning drafted the manuscript. All authors critically revised the manuscript, approved its final version and agree to be accountable for all aspects of the work in ensuring that questions related to the accuracy or integrity of any part of the work are appropriately investigated and resolved. All persons designated as authors qualify for authorship, and all those who qualify for authorship are listed.

## CONFLICT OF INTEREST

The authors declare no conflicts of interest.

## GENERATIVE AI STATEMENT

 No generative AI tools were used in the preparation of this manuscript.

## Data Availability

The data that support the findings of this study are available from the corresponding author upon reasonable request.

## References

[eph70361-bib-0001] Alari‐Pahissa, E. , Notario, L. , Lorente, E. , Vega‐Ramos, J. , Justel, A. , López, D. , Villadangos, J. A. , & Lauzurica, P. (2012). Cd69 does not affect the extent of T cell priming. PLoS ONE, 7(10), e48593. 23119065 10.1371/journal.pone.0048593 PMC3484127

[eph70361-bib-0002] Antonelli, A. , Ferri, C. , Fallahi, P. , Ferrari, S. M. , Sebastiani, M. , Ferrari, D. , Giunti, M. , Frascerra, S. , Tolari, S. , Franzoni, F. , Galetta, F. , Marchi, S. , & Ferrannini, E. (2008). High values of CXCL10 serum levels in mixed cryoglobulinemia associated with hepatitis C infection. The American Journal of Gastroenterology, 103(10), 2488–2494. 18775023 10.1111/j.1572-0241.2008.02040.x

[eph70361-bib-0064] Antonelli, A. , Ferrari, S. M. , Giuggioli, D. , Ferrannini, E. , Ferri, C. , & Fallahi, P. (2014). Chemokine (C‐X‐C motif) ligand (CXCL)10 in autoimmune diseases. Autoimmunity Reviews, 13(3), 272–280. 24189283 10.1016/j.autrev.2013.10.010

[eph70361-bib-0003] Banchereau, J. , & Steinman, R. M. (1998). Dendritic cells and the control of immunity. Nature, 392(6673), 245–252. 9521319 10.1038/32588

[eph70361-bib-0004] Brockman, M. A. , Kwon, D. S. , Tighe, D. P. , Pavlik, D. F. , Rosato, P. C. , Sela, J. , Porichis, F. , Le Gall, S. , Waring, M. T. , Moss, K. , Jessen, H. , Pereyra, F. , Kavanagh, D. G. , Walker, B. D. , & Kaufmann, D. E. (2009). Il‐10 is up‐regulated in multiple cell types during viremic HIV infection and reversibly inhibits virus‐specific T cells. Blood, 114(2), 346–356. 19365081 10.1182/blood-2008-12-191296 PMC2714209

[eph70361-bib-0005] Chesebro, B. , Bloom, M. , Wehrly, K. , & Nishio, J. (1979). Persistence of infectious Friend virus in spleens of mice after spontaneous recovery from virus‐induced erythroleukemia. Journal of Virology, 32(3), 832–837. 292801 10.1128/jvi.32.3.832-837.1979 PMC525931

[eph70361-bib-0006] Chomczynski, P. , & Sacchi, N. (1987). Single‐step method of RNA isolation by acid guanidinium thiocyanate‐phenol‐chloroform extraction. Analytical Biochemistry, 162(1), 156–159. 2440339 10.1006/abio.1987.9999

[eph70361-bib-0007] Cramer, T. , & Johnson, R. S. (2003). A novel role for the hypoxia inducible transcription factor HIF‐1alpha: Critical regulation of inflammatory cell function. Cell Cycle, 2(3), 191–192. 12734422

[eph70361-bib-0008] Cramer, T. , Yamanishi, Y. , Clausen, B. E. , Förster, I. , Pawlinski, R. , Mackman, N. , Haase, V. H. , Jaenisch, R. , Corr, M. , Nizet, V. , Firestein, G. S. , Gerber, H. P. , Ferrara, N. , & Johnson, R. S. (2003). Hif‐1alpha is essential for myeloid cell‐mediated inflammation. Cell, 112(5), 645–657. 12628185 10.1016/s0092-8674(03)00154-5 PMC4480774

[eph70361-bib-0009] Cravens, P. D. , & Lipsky, P. E. (2002). Dendritic cells, chemokine receptors and autoimmune inflammatory diseases. Immunology and Cell Biology, 80(5), 497–505. 12225387 10.1046/j.1440-1711.2002.01118.x

[eph70361-bib-0010] de Veer, M. J. , Holko, M. , Frevel, M. , Walker, E. , Der, S. , Paranjape, J. M. , Silverman, R. H. , & Williams, B. R. G. (2001). Functional classification of interferon‐stimulated genes identified using microarrays. Journal of Leukocyte Biology, 69(6), 912–920. 11404376

[eph70361-bib-0011] de Vries, J. E. (1995). Immunosuppressive and anti‐inflammatory properties of interleukin 10. Annals of Medicine, 27(5), 537–541. 8541028 10.3109/07853899509002465

[eph70361-bib-0012] Dietze, K. K. , Zelinskyy, G. , Gibbert, K. , Schimmer, S. , Francois, S. , Myers, L. , Sparwasser, T. , Hasenkrug, K. J. , & Dittmer, U. (2011). Transient depletion of regulatory T cells in transgenic mice reactivates virus‐specific CD8^+^ T cells and reduces chronic retroviral set points. Proceedings of the National Academy of Sciences of the United States of America, 108(6), 2420–2425. 21262821 10.1073/pnas.1015148108 PMC3038736

[eph70361-bib-0013] Dittmer, U. , Brooks, D. M. , & Hasenkrug, K. J. (1998). Characterization of a live‐attenuated retroviral vaccine demonstrates protection via immune mechanisms. Journal of Virology, 72(8), 6554–6558. 9658099 10.1128/jvi.72.8.6554-6558.1998 PMC109828

[eph70361-bib-0014] Dittmer, U. , Race, B. , Peterson, K. E. , Stromnes, I. M. , Messer, R. J. , & Hasenkrug, K. J. (2002). Essential roles for CD8+ T cells and gamma interferon in protection of mice against retrovirus‐induced immunosuppression. Journal of Virology, 76(1), 450–454. 11739713 10.1128/JVI.76.1.450-454.2002 PMC135723

[eph70361-bib-0015] Gabrilovich, D. I. , & Nagaraj, S. (2009). Myeloid‐derived suppressor cells as regulators of the immune system. Nature Reviews Immunology, 9(3), 162–174. 10.1038/nri2506 PMC2828349 19197294

[eph70361-bib-0016] Gabrilovich, D. I. , Ostrand‐Rosenberg, S. , & Bronte, V. (2012). Coordinated regulation of myeloid cells by tumours. Nature Reviews Immunology, 12(4), 253–268. 10.1038/nri3175 PMC3587148 22437938

[eph70361-bib-0017] Halemano, K. , Harper, M. S. , Guo, K. , Li, S. X. , Heilman, K. J. , Barrett, B. S. , & Santiago, M. L. (2013). Humoral immunity in the Friend retrovirus infection model. Immunologic Research, 55(1‐3), 249–260. 22961660 10.1007/s12026-012-8370-y PMC4003891

[eph70361-bib-0063] Harrington, L. E. , Galvan, M. , Baum, L. G. , Altman, J. D. , & Ahmed, R. (2000). Differentiating between memory and effector CD8 T cells by altered expression of cell surface O‐glycans. The Journal of Experimental Medicine, 191(7), 1241–1246. 10748241 10.1084/jem.191.7.1241 PMC2193165

[eph70361-bib-0018] Harvey, C. E. , Post, J. J. , Palladinetti, P. , Freeman, A. J. , Ffrench, R. A. , Kumar, R. K. , Marinos, G. , & Lloyd, A. R. (2003). Expression of the chemokine IP‐10 (CXCL10) by hepatocytes in chronic hepatitis C virus infection correlates with histological severity and lobular inflammation. Journal of Leukocyte Biology, 74(3), 360–369. 12949239 10.1189/jlb.0303093

[eph70361-bib-0019] Hasenkrug, K. J. (1999). Lymphocyte deficiencies increase susceptibility to friend virus‐induced erythroleukemia in Fv‐2 genetically resistant mice. Journal of Virology, 73(8), 6468–6473. 10400741 10.1128/jvi.73.8.6468-6473.1999 PMC112728

[eph70361-bib-0020] Hasenkrug, K. J. , Brooks, D. M. , Robertson, M. N. , Srinivas, R. V. , & Chesebro, B. (1998). Immunoprotective determinants in friend murine leukemia virus envelope protein. Virology, 248(1), 66–73. 9705256 10.1006/viro.1998.9264

[eph70361-bib-0021] Hasenkrug, K. J. , & Chesebro, B. (1997). Immunity to retroviral infection: The Friend virus model. Proceedings of the National Academy of Sciences of the United States of America, 94(15), 7811–7816. 9223268 10.1073/pnas.94.15.7811 PMC33712

[eph70361-bib-0022] Hasenkrug, K. J. , & Dittmer, U. (2007). Immune control and prevention of chronic Friend retrovirus infection. Frontiers in Bioscience: A Journal and Virtual Library, 12, 1544–1551. 17127401 10.2741/2167

[eph70361-bib-0023] Hegde, S. , Ni, S. , He, S. , Yoon, D. , Feng, G. S. , Watowich, S. S. , Paulson, R. F. , & Hankey, P. A. (2009). Stat3 promotes the development of erythroleukemia by inducing Pu.1 expression and inhibiting erythroid differentiation. Oncogene, 28(38), 3349–3359. 19581930 10.1038/onc.2009.202 PMC3086737

[eph70361-bib-0024] Hoatlin, M. E. , & Kabat, D. (1995). Host‐range control of a retroviral disease: Friend erythroleukemia. Trends in Microbiology, 3(2), 51–57. 7728385 10.1016/s0966-842x(00)88875-7

[eph70361-bib-0025] Hubbi, M. E. , Gilkes, D. M. , Rey, S. , Wong, C. C. , Luo, W. , Kim, D.‐H. , Dang, C. V. , Levchenko, A. , & Semenza, G. L. (2013). A nontranscriptional role for HIF‐1α as a direct inhibitor of DNA replication. Science Signaling, 6(262), ra10. 23405012 10.1126/scisignal.2003417 PMC4124626

[eph70361-bib-0026] Kabat, D. (1989). Molecular biology of Friend viral erythroleukemia. Current Topics in Microbiology and Immunology, 148, 1–42. 2684547 10.1007/978-3-642-74700-7_1

[eph70361-bib-0027] Kawaguchi, A. , & Nagata, K. (2007). De novo replication of the influenza virus RNA genome is regulated by DNA replicative helicase, MCM. The EMBO Journal, 26(21), 4566–4575. 17932485 10.1038/sj.emboj.7601881 PMC2063485

[eph70361-bib-0028] Kilani, M. M. , Mohammed, K. A. , Nasreen, N. , Tepper, R. S. , & Antony, V. B. (2004). Rsv causes HIF‐1alpha stabilization via NO release in primary bronchial epithelial cells. Inflammation, 28(5), 245–251. 16133997 10.1007/s10753-004-6047-y

[eph70361-bib-0029] Levesque, M. C. , Moody, M. A. , Hwang, K.‐K. , Marshall, D. J. , Whitesides, J. F. , Amos, J. D. , Gurley, T. C. , Allgood, S. , Haynes, B. B. , Vandergrift, N. A. , Plonk, S. , Parker, D. C. , Cohen, M. S. , Tomaras, G. D. , Goepfert, P. A. , Shaw, G. M. , Schmitz, J. E. , Eron, J. J. , Shaheen, N. J. , … B, F. (2009). Polyclonal B cell differentiation and loss of gastrointestinal tract germinal centers in the earliest stages of HIV‐1 infection. PLoS Medicine, 6(7), e1000107. 19582166 10.1371/journal.pmed.1000107 PMC2702159

[eph70361-bib-0030] Lilly, F. (1968). The effect of histocompatibility‐2 type on response to friend leukemia virus in mice. The Journal of Experimental Medicine, 127(3), 465–473. 5636554 10.1084/jem.127.3.465 PMC2138461

[eph70361-bib-0031] Lilly, F. , & Steeves, R. A. (1973). B‐tropic Friend virus: A host‐range pseudotype of spleen focus‐forming virus (SFFV). Virology, 55(2), 363–370. 4742777 10.1016/0042-6822(73)90176-1

[eph70361-bib-0032] Liu, B. , Tonkonogy, S. L. , & Sartor, R. B. (2011). Antigen‐presenting cell production of IL‐10 inhibits T‐helper 1 and 17 cell responses and suppresses colitis in mice. Gastroenterology, 141(2), 653–662.e4. 21679711 10.1053/j.gastro.2011.04.053 PMC4651012

[eph70361-bib-0033] Liu, M. , Guo, S. , Hibbert, J. M. , Jain, V. , Singh, N. , Wilson, N. O. , & Stiles, J. K. (2011). Cxcl10/ip‐10 in infectious diseases pathogenesis and potential therapeutic implications. Cytokine & Growth Factor Reviews, 22(3), 121–130. 21802343 10.1016/j.cytogfr.2011.06.001 PMC3203691

[eph70361-bib-0034] Livak, K. J. , & Schmittgen, T. D. (2001). Analysis of relative gene expression data using real‐time quantitative PCR and the 2(‐Delta Delta C(T)) Method. Methods, 25(4), 402–408. 11846609 10.1006/meth.2001.1262

[eph70361-bib-0062] Lord, S. J. , Rajotte, R. V. , Korbutt, G. S. , & Bleackley, R. C. (2003). Granzyme B: A natural born killer. Immunological Reviews, 193, 31–38. 12752668 10.1034/j.1600-065x.2003.00044.x

[eph70361-bib-0035] Marcelletti, J. , & Furmanski, P. (1979). Infection of macrophages with Friend virus: Relationship to the spontaneous regression of viral erythroleukemia. Cell, 16(3), 649–659. 287567 10.1016/0092-8674(79)90038-2

[eph70361-bib-0036] Moreau‐Gachelin, F. (2008). Multi‐stage Friend murine erythroleukemia: Molecular insights into oncogenic cooperation. Retrovirology, 5, 99. 18983647 10.1186/1742-4690-5-99 PMC2585586

[eph70361-bib-0037] Nagaraj, S. , Gupta, K. , Pisarev, V. , Kinarsky, L. , Sherman, S. , Kang, L. , Herber, D. L. , Schneck, J. , & Gabrilovich, D. I. (2007). Altered recognition of antigen is a mechanism of CD8^+^ T cell tolerance in cancer. Nature Medicine, 13(7), 828–835. 10.1038/nm1609 PMC2135607 17603493

[eph70361-bib-0038] Nair, S. R. , Zelinskyy, G. , Schimmer, S. , Gerlach, N. , Kassiotis, G. , & Dittmer, U. (2010). Mechanisms of control of acute Friend virus infection by CD4^+^ T helper cells and their functional impairment by regulatory T cells. The Journal of General Virology, 91(Pt 2), 440–451. 19828756 10.1099/vir.0.015834-0

[eph70361-bib-0039] Nakayama, T. , Kasprowicz, D. J. , Yamashita, M. , Schubert, L. A. , Gillard, G. , Kimura, M. , Didierlaurent, A. , Koseki, H. , & Ziegler, S. F. (2002). The generation of mature, single‐positive thymocytes in vivo is dysregulated by CD69 blockade or overexpression. Journal of Immunology, 168(1), 87–94. 10.4049/jimmunol.168.1.87 11751950

[eph70361-bib-0040] Naldini, A. , Carraro, F. , Fleischmann, W. R. , & Bocci, V. (1993). Hypoxia enhances the antiviral activity of interferons. Journal of Interferon Research, 13(2), 127–132. 8389791 10.1089/jir.1993.13.127

[eph70361-bib-0041] Norris, B. A. , Uebelhoer, L. S. , Nakaya, H. I. , Price, A. A. , Grakoui, A. , & Pulendran, B. (2013). Chronic but not acute virus infection induces sustained expansion of myeloid suppressor cell numbers that inhibit viral‐specific T cell immunity. Immunity, 38(2), 309–321. 23438822 10.1016/j.immuni.2012.10.022 PMC3869405

[eph70361-bib-0042] Robertson, M. N. , Miyazawa, M. , Mori, S. , Caughey, B. , Evans, L. H. , Hayes, S. F. , & Chesebro, B. (1991). Production of monoclonal antibodies reactive with a denatured form of the Friend murine leukemia virus gp70 envelope protein: Use in a focal infectivity assay, immunohistochemical studies, electron microscopy and western blotting. Journal of Virological Methods, 34(3), 255–271. 1744218 10.1016/0166-0934(91)90105-9

[eph70361-bib-0043] Robertson, M. N. , Spangrude, G. J. , Hasenkrug, K. , Perry, L. , Nishio, J. , Wehrly, K. , & Chesebro, B. (1992). Role and specificity of T‐cell subsets in spontaneous recovery from Friend virus‐induced leukemia in mice. Journal of Virology, 66(6), 3271–3277. 1374804 10.1128/jvi.66.6.3271-3277.1992 PMC241104

[eph70361-bib-0044] Santos, S. , Obukhov, Y. , Nekhai, S. , Pushkarsky, T. , Brichacek, B. , Bukrinsky, M. , & Iordanskiy, S. (2016). Cellular minichromosome maintenance complex component 5 (MCM5) is incorporated into HIV‐1 virions and modulates viral replication in the newly infected cells. Virology, 497, 11–22. 27414250 10.1016/j.virol.2016.06.023 PMC5079758

[eph70361-bib-0045] Saraiva, M. , & O'Garra, A. (2010). The regulation of IL‐10 production by immune cells. Nature Reviews Immunology, 10(3), 170–181. 10.1038/nri2711 20154735

[eph70361-bib-0046] Schmid, M. A. , Kingston, D. , Boddupalli, S. , & Manz, M. G. (2010). Instructive cytokine signals in dendritic cell lineage commitment. Immunological Reviews, 234(1), 32–44. 20193010 10.1111/j.0105-2896.2009.00877.x

[eph70361-bib-0047] Sugamura, K. , Ishii, N. , & Weinberg, A. D. (2004). Therapeutic targeting of the effector T‐cell co‐stimulatory molecule OX40. Nature Reviews Immunology, 4(6), 420–431. 10.1038/nri1371 15173831

[eph70361-bib-0048] Toniolo, A. , Matteucci, D. , Pistillo, M. P. , Gori, Z. , & Bendinelli, M. (1980). Early replication of Friend leukaemia viruses in spleen macrophages. The Journal of General Virology, 49(1), 203–208. 7420064 10.1099/0022-1317-49-1-203

[eph70361-bib-0049] Vollbrecht, T. , Stirner, R. , Tufman, A. , Roider, J. , Huber, R. M. , Bogner, J. R. , Lechner, A. , Bourquin, C. , & Draenert, R. (2012). Chronic progressive HIV‐1 infection is associated with elevated levels of myeloid‐derived suppressor cells. AIDS, 26(12), F31–F37. 22526518 10.1097/QAD.0b013e328354b43f

[eph70361-bib-0050] Wang, G. L. , Jiang, B. H. , Rue, E. A. , & Semenza, G. L. (1995). Hypoxia‐inducible factor 1 is a basic‐helix‐loop‐helix‐PAS heterodimer regulated by cellular O2 tension. Proceedings of the National Academy of Sciences of the United States of America, 92(12), 5510–5514. 7539918 10.1073/pnas.92.12.5510 PMC41725

[eph70361-bib-0051] Weissman, I. L. (2002). The road ended up at stem cells. Immunological Reviews, 185, 159–174. 12190929 10.1034/j.1600-065x.2002.18514.x

[eph70361-bib-0052] Wenger, R. H. (2002). Cellular adaptation to hypoxia: O2‐sensing protein hydroxylases, hypoxia‐inducible transcription factors, and O2‐regulated gene expression. FASEB Journal : Official Publication of the Federation of American Societies for Experimental Biology, 16(10), 1151–1162. 12153983 10.1096/fj.01-0944rev

[eph70361-bib-0053] Wherry, E. J. , Ha, S. ‑ J. , Kaech, S. M. , Haining, W. N. , Sarkar, S. , Kalia, V. , Subramaniam, S. , Blattman, J. N. , Barber, D. L. , & Ahmed, R. (2007). Molecular signature of CD8^+^ T cell exhaustion during chronic viral infection. Immunity, 27(4), 670–684. 17950003 10.1016/j.immuni.2007.09.006

[eph70361-bib-0054] Wilson, E. B. , & Brooks, D. G. (2011). The role of IL‐10 in regulating immunity to persistent viral infections. Current Topics in Microbiology and Immunology, 350, 39–65. 20703965 10.1007/82_2010_96 PMC3492216

[eph70361-bib-0055] Wobben, R. , Hüsecken, Y. , Lodewick, C. , Gibbert, K. , Fandrey, J. , & Winning, S. (2013). Role of hypoxia inducible factor‐1α for interferon synthesis in mouse dendritic cells. Biological Chemistry, 394(4), 495–505. 23362200 10.1515/hsz-2012-0320

[eph70361-bib-0056] Yoneyama, H. , Narumi, S. , Zhang, Y. , Murai, M. , Baggiolini, M. , Lanzavecchia, A. , Ichida, T. , Asakura, H. , & Matsushima, K. (2002). Pivotal role of dendritic cell‐derived CXCL10 in the retention of T helper cell 1 lymphocytes in secondary lymph nodes. The Journal of Experimental Medicine, 195(10), 1257–1266. 12021306 10.1084/jem.20011983 PMC2193754

[eph70361-bib-0057] Zelinskyy, G. , Dietze, K. , Sparwasser, T. , & Dittmer, U. (2009). Regulatory T cells suppress antiviral immune responses and increase viral loads during acute infection with a lymphotropic retrovirus. PLoS Pathogens, 5(8), e1000406. 19714239 10.1371/journal.ppat.1000406 PMC2727466

[eph70361-bib-0058] Zelinskyy, G. , Kraft, A. R. M. , Schimmer, S. , Arndt, T. , & Dittmer, U. (2006). Kinetics of CD8+ effector T cell responses and induced CD4^+^ regulatory T cell responses during Friend retrovirus infection. European Journal of Immunology, 36(10), 2658–2670. 16981182 10.1002/eji.200636059

[eph70361-bib-0059] Zelinskyy, G. , Robertson, S. J. , Schimmer, S. , Messer, R. J. , Hasenkrug, K. J. , & Dittmer, U. (2005). Cd8^+^ T‐cell dysfunction due to cytolytic granule deficiency in persistent Friend retrovirus infection. Journal of Virology, 79(16), 10619–10626. 16051854 10.1128/JVI.79.16.10619-10626.2005 PMC1182617

[eph70361-bib-0060] Zhang, N. , & Bevan, M. J. (2010). Dicer controls CD8^+^ T‐cell activation, migration, and survival. Proceedings of the National Academy of Sciences of the United States of America, 107(50), 21629–21634. 21098294 10.1073/pnas.1016299107 PMC3003005

[eph70361-bib-0061] Zinkernagel, A. S. , Johnson, R. S. , & Nizet, V. (2007). Hypoxia inducible factor (HIF) function in innate immunity and infection. Journal of Molecular Medicine, 85(12), 1339–1346. 18030436 10.1007/s00109-007-0282-2

